# Optimizing IABP–patient interaction in VA-ECMO via transcranial doppler

**DOI:** 10.1186/s13089-025-00460-2

**Published:** 2025-12-05

**Authors:** Andres Felipe Yepes-Velasco, Jeimy Lorena Moreno-Araque, Natalia Garzon-Posada

**Affiliations:** 1 Department of Critical Medicine and Intensive Care, Fundación Santa Fe De Bogotá, Carrera 7 No. 117 - 15, Bogotá, Colombia; 2 School of Medicine and Health Sciences, Universidad Del Rosario, Bogotá, Colombia

**Keywords:** Transcranial Doppler, Veno-arterial extracorporeal membrane oxygenation, Intra-aortic balloon pump, Cerebral perfusion, Neurosonology, Critical care ultrasound, Patient–device interaction

## Abstract

**Background:**

Patients supported with veno-arterial extracorporeal membrane oxygenation may receive an intra-aortic balloon pump to reduce left ventricular afterload and improve aortic diastolic pressure. However, the effect of this combined mechanical support on cerebral hemodynamics is not uniform and can be influenced by intra-aortic balloon pump timing. Bedside transcranial Doppler offers a rapid, noninvasive way to detect maladaptive cerebral flow patterns and to guide patient–device interaction in real time.

**Case presentation:**

We describe a postcardiotomy adult patient on peripheral veno-arterial extracorporeal membrane oxygenation with concomitant intra-aortic balloon pump assistance (1:1) who developed a reduction in cerebral oximetry. Transcranial Doppler of the middle cerebral artery showed increased pulsatility and reduced diastolic velocity, findings consistent with a transient decrease in cerebral perfusion pressure and compatible with balloon deflation asynchrony. Temporary suspension of balloon assistance improved the waveform. Deflation was then synchronized with the electrocardiogram so that it was completed at the onset of systole. Repeat transcranial Doppler performed minutes later showed restoration of diastolic flow and a lower pulsatility index, while extracorporeal support was maintained unchanged and the patient remained hemodynamically stable.

**Conclusions:**

In patients receiving veno-arterial extracorporeal membrane oxygenation and intra-aortic balloon pump support, cerebral blood flow may deteriorate if balloon timing is not aligned with the native cardiac cycle. Transcranial Doppler can detect these timing-related neurohemodynamic alterations at the bedside and can confirm their reversibility after simple, ECG-guided optimization of deflation. Integrating transcranial Doppler into multiparametric monitoring may help personalize mechanical circulatory support and protect cerebral perfusion in this high-risk population.

## Background

Veno-arterial extracorporeal membrane oxygenation (VA-ECMO) is a temporary circulatory mechanical support used for severe cardiogenic shock or cardiac arrest to restore systemic perfusion while gas exchange occurs extracorporeally [1, 2]. Because VA-ECMO does not fully unload the left ventricle, adjunct strategies are often required. In this setting, the intra-aortic balloon pump (IABP) is frequently used to reduce effective arterial elastance and left-ventricular afterload and to augment diastolic aortic pressure [3, 4]. Reports on cerebral hemodynamics during combined VA-ECMO + IABP are heterogeneous, reflecting variability in residual native ejection and device timing [5, 6]. 

Point-of-care neurosonography (TCD/TCCD) offers a practical, non-invasive way to trend cerebral hemodynamics at the bedside and to contextualize device interactions in patients on VA-ECMO with IABP [6–9]. We present a postcardiotomy patient with a transient reduction in cerebral perfusion detected by TCD and corrected through TCD-guided optimization of IABP timing, and we outline a focused, sequential monitoring approach for similar cases.

### Case presentation

A 62-year-old patient with a mechanical aortic valve replacement (1985) was admitted with congestive heart failure due to atrial flutter and severe mitral insufficiency. After medical stabilization, the patient underwent mechanical mitral valve replacement with a St. Jude Regent #33 prosthesis, combined with anomalous pathway ablation, maze surgery (cryo and radiofrequency, biatrial), left atrial appendage exclusion, and intracavitary thrombus resection. The procedure had an ischemia time of 147 min and perfusion time of 190 min.

**Fig. 1:**
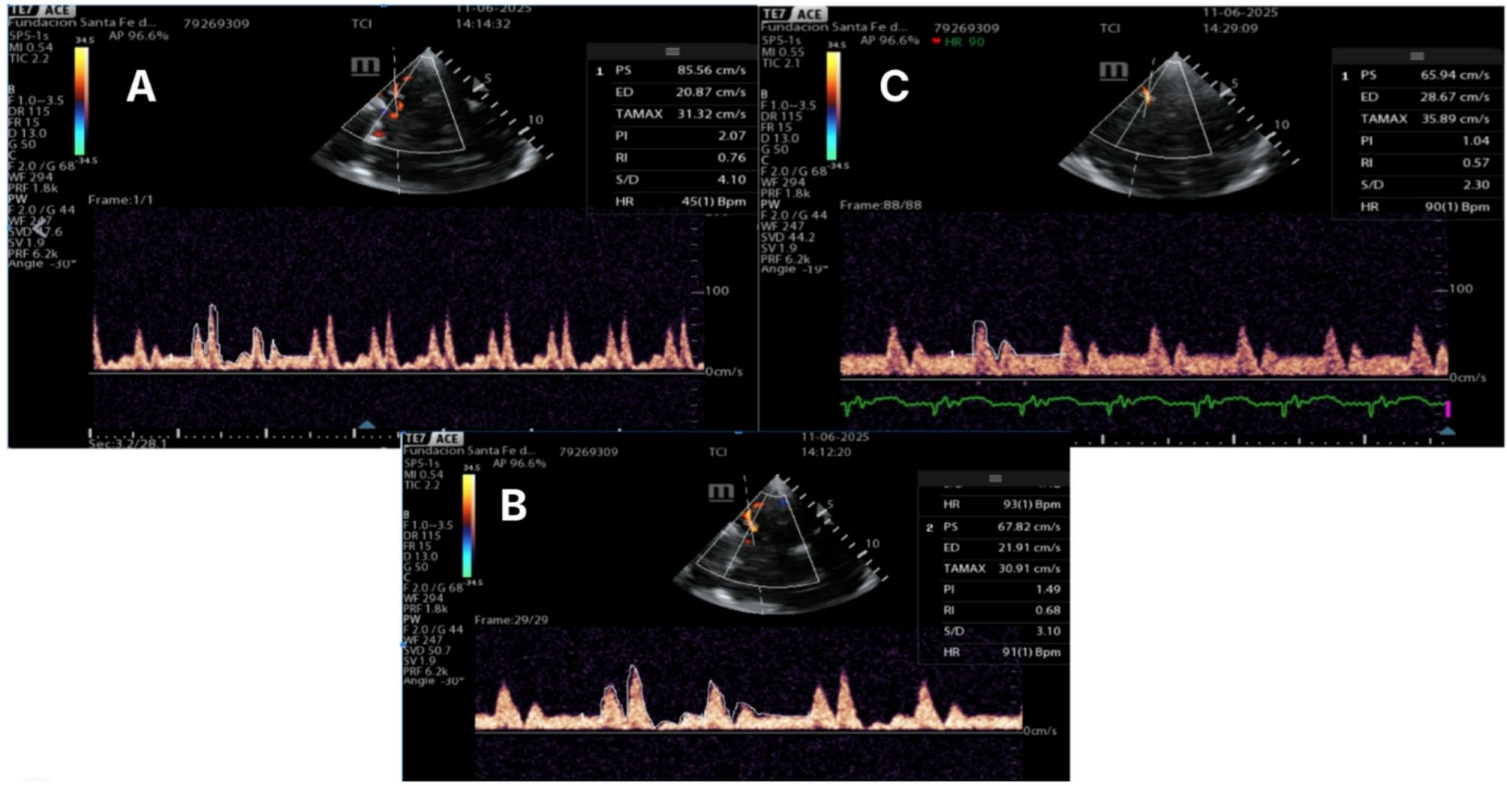
Flow of the middle cerebral artery with 1:1 assistance (**a**) 1:2 assistance (**b**) and without assistance (**c**). an increase in the pulsatility index with a drop in diastolic velocity is noted, which improves as ventricular assistance is reduced through the intra-aortic balloon pump. Two representative cardiac cycles are displayed to demonstrate beat-to-beat variability across assistance settings (IABP 1:1, 1:2, paused). Quantitative values in the text were calculated by averaging 3–5 consecutive cycles under the same condition

Postoperatively, the patient developed severe oxygenation impairment and refractory hemodynamic deterioration, requiring peripheral veno-arterial femoro-femoral ECMO and intra-aortic balloon pump (IABP) support. During bedside monitoring with the IABP synchronized at 1:1 and ECMO flow at 4.2 L, a reduction in bilateral cerebral oximetry (NIRS) was observed. Transcranial Doppler revealed a low-flow, high-resistance cerebral pattern (Fig. 1), and the echocardiogram showed severely reduced systolic function LVEF < 30%.

**Fig. 2:**
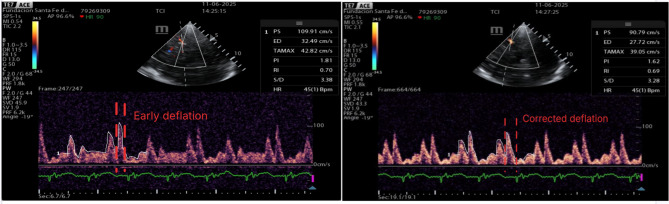
Changes in middle cerebral artery flow velocities after manually adjusting the intra-aortic balloon pump timing, specifically adjusting the deflation time to occur at the beginning of the QRS complex on the ECG, allowing the deflation to continue during early systole. A single representative cycle is shown to emphasize timing landmarks (deflation onset at the QRS) without visual clutter. Quantitative interpretation was based on 3–5-cycle averaging recorded during the same condition

When IABP assistance was temporarily paused—without changing ECMO flow or fresh gas settings—immediate improvements in pulsatility indices and flow were observed (Fig. 1). These findings suggested a transient cerebral hypoperfusion pattern (characterized by increased pulsatility index with reduced diastolic velocity) that promptly normalized following ECG-gated optimization of IABP timing parameters. (Fig. 2).

## Methods for neurosonography and interpretation

Bedside TCD/TCCD examinations of the MCA were performed via the transtemporal window (typical depth 50–55 mm; sample volume 5–7 mm) with angle correction as needed. Sweep speed was adjusted to visualize multiple cardiac cycles (approximately 3–6 s per screen). For quantitative interpretation, we averaged 3–5 consecutive cycles free of ectopy/artifact at a stable heart rate. Device settings (IABP assistance ratio and timing; VA-ECMO flow and gas) were documented for each acquisition. Figure panels may display 1–2 representative beats solely for visual clarity (e.g., to illustrate beat-to-beat variability under changing assistance, or to mark precise timing landmarks such as QRS-aligned deflation). No numerical values were derived from a single beat; all reported velocities and PI reflect 3–5-beat averaging from the same condition.

### Bedside integration

For patients on VA-ECMO + IABP, we suggest this pragmatic sequence:

1. Interrogate MCA flow with TCD/TCCD, standardizing acquisition (appropriate depth, angle correction as needed, sweep speed showing several cycles). Use 3–5-beat averaging for quantitative interpretation, while selectively using 1–2 displayed beats to highlight beat-to-beat variability or precise timing landmarks [10, 11].
2. Optimize IABP timing: ensure early diastolic inflation and rapid deflation completed before systole (QRS-gated when needed). Reassess via TCD within minutes to confirm improvement in diastolic velocity and reduction in maladaptive PI trends.
3. Integrate TCD into the multiparametric evaluation for optimizing patient-device interaction.

## Discussion and Conclusion

Neurological complications during VA-ECMO are multifactorial, stemming from pre-support disease severity, arrest circumstances, ECMO-induced metabolic alterations (especially sudden PaCO₂ changes), residual cardiac function, and supplementary devices [12–14]. Neuromonitoring in these scenarios is crucial for early detection of associated neurological complications and guiding therapeutic interventions. In our case, we observed a temporary decrease in middle cerebral artery (MCA) diastolic flow and elevated pulsatility during 1:1 IABP assistance. This hemodynamic pattern was optimized through careful adjustment of IABP ECG-gated synchronization.

The interplay between cardiac function, cerebral perfusion, and intra-aortic balloon pump (IABP) support is mediated chiefly by reductions in effective arterial elastance and left-ventricular afterload with diastolic augmentation; the accompanying rise in cardiac output is typically modest (~ 0.5–1.0 L/min) [5, 6]. At the cerebral level, the balance between diastolic augmentation and systolic impedance shapes the middle cerebral artery (MCA) waveform on TCD. It has been reported—in a small, hypothesis-generating cohort—that during myocardial stunning with very low pulse pressure (PP < 10 mmHg), counterpulsation may reduce cerebral blood flow due to unfavorable timing and limited forward stroke volume; conversely, with partial recovery (PP ≥ 10 mmHg), IABP may increase cerebral flow relative to VA-ECMO alone. Given the small sample size and potential confounders, these observations should not be taken as prescriptive; rather, they reinforce the need for TCD monitoring [15]. This suggests that the cerebral effect of IABP during VA-ECMO is state-dependent and hinges on timing. This is particularly relevant in patients with severe heart failure, where cerebral autoregulation capacity is compromised and cerebral blood flow may be reduced [16].

Ultrasound can be invaluable in the assessment of patients on ECMO, particularly due to the complexity of transporting these patients for diagnostic imaging, allowing for integrated multi-organ monitoring [7]. Transcranial Doppler has been utilized in various clinical scenarios, including the characterization of cerebral perfusion pressure, evaluation of brain death and other neurological assessments, it provides rapid readouts of diastolic velocity and pulsatility trends, captures beat-to-beat responses to timing changes, integrates with NIRS, and can be repeated safely at the bedside where transport is risky [8, 9]. This role is consistent with international recommendations positioning ultrasound-based neuromonitoring as a core ICU competency [10, 11]. The pulsatility index (PI) is not a pure surrogate of distal cerebrovascular resistance; it reflects the interplay among cerebral perfusion pressure, arterial pulse amplitude, vascular compliance and tone, cardiac function and heart rate. Accordingly, PI should be interpreted contextually—with MAP, pulse pressure, and device settings—rather than as a stand-alone estimate of intracranial pressure [17]. In our patient, the maladaptive neurohemodynamic pattern—manifested by rising PI and reduced diastolic velocities—is most consistent with a temporary drop in CPP. Although diastolic reversal was not documented, the observed changes are coherent with a reversal tendency reported in IABP when deflation is delayed [18], and they improved after ECG-gated synchronization.

Clinically, the question is seldom “IABP on vs off,” but how the balloon is timed relative to the native cardiac cycle. Inflation should occur at diastole onset to augment aortic diastolic pressure, and deflation should be completed just before systole (often triggered at the QRS) to lower afterload and facilitate forward flow. Suboptimal deflation (too late or slow) may elevate systolic impedance, worsening cerebral perfusion despite adequate mean arterial pressure. In our patient, re-timing deflation restored diastolic augmentation and improved MCA diastolic velocity within minutes. This illustrates how minute-to-minute titration of assistance, guided by TCD, can optimize patient device interaction.

## Limitations

This is a single-patient, real-world observation without protocolized hemodynamic targets or invasive intracranial monitoring. TCD measures were interpreted alongside clinical variables, acknowledging that PI reflects multiple determinants. Images were curated for teaching clarity; going forward, we have standardized sweep speed and 3–5-cycle averaging in our protocol to enhance reproducibility.

## Data Availability

No datasets were generated or analysed during the current study.
